# Efficacy of Mobile Health Applications to Improve Physical Activity and Sedentary Behavior: A Systematic Review and Meta-Analysis for Physically Inactive Individuals

**DOI:** 10.3390/ijerph19084905

**Published:** 2022-04-18

**Authors:** Meng Zhang, Wei Wang, Mingye Li, Haomin Sheng, Yifei Zhai

**Affiliations:** 1 Department of Physical Education, Nanjing University, 163 Xianlin Road, Qixia District, Nanjing 210023, China; 2 Department of Software Systems and Cybersecurity, Faculty of Information Technology, Monash University, Clayton 3800, Australia; wei.wang5@monash.edu; 3 School of Computing and Information Systems, Faculty of Engineering and Information Technology, The University of Melbourne, Parkville 3010, Australia; mingye.li1@unimelb.edu.au; 4 Department of Information Systems and Business Analytics, College of Business and Law, RMIT University, Melbourne 3001, Australia; 5 School of Intellectual Property, Nanjing University of Science and Technology, Nanjing 210094, China; 19962011352@163.com

**Keywords:** physically inactive people, meta-analysis, mobile health, physical activity, sedentary behavior

## Abstract

Physical inactivity and sedentary behavior (SB) have attracted growing attention globally since they relate to noninfectious chronic diseases (NCDs) and could further result in the loss of life. This systematic literature review aimed to identify existing evidence on the efficacy of mobile health (mHealth) technology in inducing physical activity and reducing sedentary behavior for physically inactive people. Studies were included if they used a smartphone app in an intervention to improve physical activity and/or sedentary behavior for physically inactive individuals. Interventions could be stand-alone interventions or multi-component interventions, including an app as one of several intervention components. A total of nine studies were included, and all were randomized controlled trials. Two studies involved interventions delivered solely via a mobile application (stand-alone intervention) and seven studies involved interventions that used apps and other intervention strategies (multi-component intervention). Methodological quality was assessed, and the overall quality of the studies was ensured. The pooled data favored intervention in improving physical activity and reducing sedentary behavior. This review provided evidence that mobile health intervention improved physical activity and reduced sedentary behavior among inactive individuals. More beneficial effects can be guaranteed when interventions include multiple components. Further studies that maintain the effectiveness of such interventions are required to maximize user engagement and intervention efficacy.

## 1. Introduction

Regular physical activity (PA) could promote individual physical and mental health [1]. The World Health Organization (WHO) and the American College of Sports Medicine (ACSM) advocate that “*adults aged 18–64 should do at least 150 min of moderate-intensity aerobic PA or do at least 75 min of vigorous-intensity aerobic PA per week or an equivalent combination of both*” [2]. However, nearly 58% of individuals have not achieved the recommended amount of activity (i.e., 2.5 h per week) [3], and thus, they are considered physically inactive. In fact, physical inactivity has been deemed as the fourth driver of global mortality and the chief cause of non-communicable diseases (NCDs) [4]. Meanwhile, meta-analyses show that even those who are meeting these recommendations may still be sitting for a long while, which could lead to potential health risks associated with sedentary behavior (SB) [5,6]. Therefore, the two growingly interrelated themes, “sedentary and inactive”, have become a global health challenge faced by humans [7]. 

Health interventions based on the behavior analysis present the potentials to increase daily physical activity levels [8,9]. Therefore, it is critical to explore preventive interventions that the general population could easily follow. However, traditional face-to-face interventions in public health may not achieve such a purpose [10,11,12,13,14]. This is because people may have limited access due to internal/personal (e.g., lack of time or motivation) and external obstacles (e.g., commuting conditions and the expense of activities) [15,16]. With the development of digital technologies, the concept of mobile health (mHealth) interventions has emerged, which refers to medical and public health practice supported by mobile phones, patient-monitoring devices, Personal Digit Assistant (PDA), and other wireless devices [17].

As reported, mobile health applications have been broadly employed to gather and analyze health-related data and accordingly to design interventions that could help facilitate positive behavior changes for healthy purposes [18]. Particularly, mHealth could target intervening in physical inactivity and sedentary behaviors that require durative persistence since mHealth can offer interactive technologies (e.g., activity reminders and peer support) capable of increasing users’ adherence to the interventions [19,20]. Meanwhile, the mHealth interventions, whose designs tend to be user-friendly [9], can be easily delivered anywhere and anytime, benefiting from the broad popularity of mobile devices [21]. Thus, mHealth are expected to promote the public health care universally.

The domain of mHealth has attracted research interests from multiple disciplines such as sociology and psychology. Though several systematic reviews on mHealth interventions indicate that it is efficacious for average people in promoting behavioral conversion and improving well-being [22,23], there are still some limitations or weaknesses. On the one hand, most of the systemic reviews in the field of mHealth interventions are organized by just focusing on the mHealth application [22,23,24,25] or smartphones [26,27], mHealth interventions based on PDA or other wireless devices tend to be ignored by the former studies. On the other hand, some positive impacts of the mHealth interventions on the PA and the SB are reported [23,24,25,26,27], while the specialized discussion as well as the systematic reviews targeting the representative population—the inactive group—in the trend of the global lack of PA and the popularity of SB is thus far insufficient. These identified research problems motivate this study to target the overall formats of mHealth interventions and the specific inactive population.

Hence, this study aims to systematically review and investigate to what extent mHealth interventions can help to improve physical activity and mitigate sedentary behavior among the inactive group. It synthesizes the outcomes reported in the included randomized controlled trials (RCTs) based on the comprehension of the problems that existed in the related systematic reviews and meta-analyses on health management and support by means of the mHealth. Through the meta-analysis, this paper explores the change in the duration of PA and SB brought by mHealth interventions among the inactive group and thus proposes measures such as selecting the proper exercise plans in mHealth applications to promote individual physical activity and reduce sedentary behavior.

This paper could add value to present knowledge. Our research indicates that the inactive group tends to exhibit its unique and non-linear nature in terms of the duration of PA and SB after the intervention. In other words, there is a sharp response at the initial stage and a successively irregular declination afterward. The focus on inactive individuals could enhance the specificity of current studies on mHealth interventions. As a result, more reliable suggestions for the improvement in mHealth interventions particularly targeting the inactive group could be proposed. We could thus expect to observe some beneficial changes such as being less sedentary and more physically active among inactive individuals by following our research results and arousing their cognition of seeking effective support from mHealth interventions.

## 2. Method 

The Preferred Reporting Items for Systematic Reviews and Meta-Analyses (PRISMA) Guidelines and Cochrane Handbook for Systematic Reviews of Interventions were used as methodological templates for this review [28].

### 2.1. Information Sources and Search Strategy

Literature searches were conducted in March 2022. Five databases were searched, including Web of Science, CINAHL, Scopus, PsychInfo, and PubMed. The sources were limited to peer-reviewed journal articles published between 2006 and 2022. It was considered unlikely that mobile health interventions were included before 2006 when smartphones were introduced. To organize and construct the search string(s), we follow the PICO approach [29]: population, intervention, comparison, and outcomes of interest (see Table 1). Two concepts were selected to develop the search query (Table 2). Searched digital libraries and the corresponding search string(s) used are shown in Table 3. Searches followed the PRISMA statement targeting the following keywords in the title or abstract. 

The search results were imported into Mendeley bibliographic software. Duplicate studies were removed. The titles and abstracts of all identified studies were screened to identify potentially relevant papers. Studies that did not meet the inclusion criteria and those whose titles/abstracts were obviously not related to the topic of interest were excluded from this review. To ensure that there was no potential for missing primary research, we also searched for those in the automatic search. Backward snowballing was conducted after screening the abstract and title. Then, full-text papers of potentially relevant studies were retrieved and assessed for eligibility by two reviewers. Where uncertainties arose regarding study inclusion, a consensus was achieved through discussion with the third reviewer.

### 2.2. Inclusion and Exclusion Criteria

Identified studies were screened for eligibility if they met the following inclusion criteria: (1) inactive population, which is the general population without disease that cannot meet the recommended standard of PA or tends to be sedentary daily; (2) published RCTs with mobile health intervention influencing at least one of the following lifestyle behaviors: PA, SB; (3) the mobile health intervention could be a stand-alone intervention or a multi-component intervention; and (4) the report of a study written by the researchers who actually performed the study.

Exclusion criteria leading to studies being classified as ineligible were: (1) age <18 years and age >64; (2) non-experimental study designs; (3) mobile technology was used to provide information versus used for self-management; and (4) clinically diagnosed populations with the exception of those who are overweight or obese.

### 2.3. Data Extraction and Risk of Bias of Included Studies

The following data were independently extracted from each paper using a standardized form: author, study design, duration, participant characteristics, intervention description, dependent variables, outcome measures, and comparison groups—similar to those used in other systematic reviews [22,30,31]. Two reviewers independently extracted data from each included study. Both reviewers one and two agreed on the data extraction in over 70% of the studies. Disagreement was easily resolved by discussion and consensus with a third reviewer.

The risk of bias assessment of the included studies was conducted by two reviewers using the Cochrane Collaboration’s risk of bias tool [32]. It considers bias originating from the following domains: (i) Random sequence generation (selection bias); (ii) allocation concealment (selection bias); (iii) blinding of participants and personal (performance bias); (iv) blinding of outcome assessment (detection bias); (v) incomplete outcome data (attrition data); (vi) selective reporting (reporting bias); and (vii) other bias. Two reviewers independently marked risk of bias level for each domain. Where inconsistency arose regarding the risk of bias, a consensus was achieved through discussion with the third reviewer.

Studies were considered low-risk of bias for blinding of the outcome assessment (domain (iv)) if the objective measures of PA and SB were used for data collection. Instead, the risk of bias in the study outcome assessment was considered high if the data collection on the outcome is based on subjective measures. For the selective reporting (domain (vi)), studies were judged to be low-risk if there were published protocol papers and the study followed the plan. In the absence of publicly available protocol papers, studies reporting all outcomes mentioned in the methodology are considered low-risk bias of selective reporting.

### 2.4. Study Quality Assessment

Twenty-five-point criteria adapted from the CONSORT checklists, which are applicable to control trail and other study designs, were used to assess the quality of the included studies [30]. This approach has been used in other reviews [22,30]. Each criterion from the CONSORT checklists was scored as 1 (fulfilled), 0.5 (not all sub-items making up the criterion were fulfilled), 0 (not fulfilled or unclear), or not applicable to the study design. A percentage of fulfilled criteria can be calculated by dividing the obtained study quality score by the highest attainable score that has been used in other reviews [22,30]. Not applicable criteria were discounted from the attainable study quality score. The rate of the included studies was categorized into high (>66.7%), mid (50–66.6%) or low (<50%) study quality. Reviewers independently scored a random half of RCTs. Then, reviewers cross-assessed 4 of 9 RCTs (44%) and reached consensus on disagreements. Reviewers reassessed the remaining studies by applying the consensus criteria.

### 2.5. Strategy for Data Synthesis and Analysis

Eight studies [21,33,34,35,36,37,38] had continuous outcomes for measures of PA across the same scale, allowing meta-analysis of mean differences (MD). The units of the PA data reported in the study were mostly minutes per day or week of varying intensity. If the study provided data on the amount of time spent on physical activity per week, these were translated into minutes per day (e.g., 420 min/week = 60 min/day), which was also used in other reviews [31,39]. Studies in which the information was unavailable or reported units could not be converted to the scale of min/day were not included in the meta-analyses [40]. If there is more than one measure of PA, objective data takes precedence over subjective data. If there is more than one objective measure of PA, preference will be given to the measure that best represents overall PA [31]. If the focus of a study is to increase vigorous PA, then vigorous PA data will be prioritized and used in the analysis. The other two meta-analyses performed for interventions reported follow-up PA and SB outcome measures. Given the small sample size, subgroup analyses were not performed.

The imported data were assessed for statistical heterogeneity. A random effects model would be adopted when the value was moderate (30% to 60%) to substantial (50% to 90%). Otherwise, a fixed effects model would be adopted. All results of the intervention group and control group were summarized by means and standard deviations.

## 3. Results

### 3.1. Search Results

**Figure 1:**
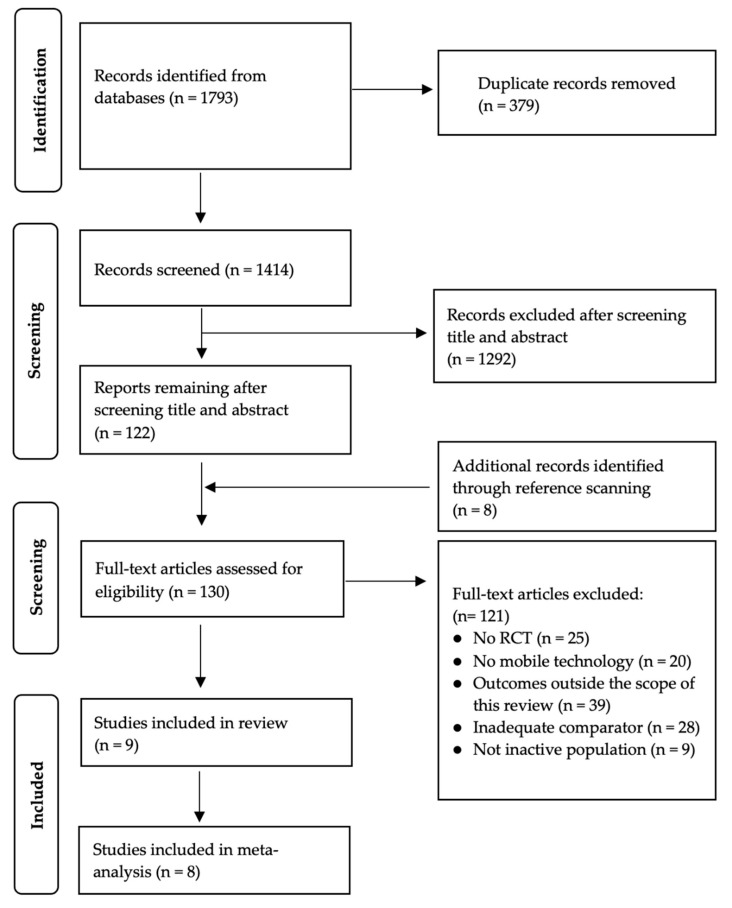
PRISMA flow diagram.

The PRISMA flow diagram of the study selection process is presented in Figure 1. A total of 1793 studies were included in the review after employing the search strategy. The number reduced to 1414 after removing 379 duplicates, which were consequently assessed against the title and abstract. A total of 1292 articles were excluded after screening the title and abstract. References of eligible studies were manually scanned to identify any additional studies and a further eight papers from our backward snowballing search. Further filtering was conducted by screening the full text of the study. Among these, 121 articles were removed since they were not considered highly related to our focus area, e.g., not RCT (*n* = 25), not using mobile technology for the intervention (*n* = 20), outcomes outside the scope of this review (*n* = 39), inadequate comparator (*n* = 28), and targeting population not inactive (*n* = 9). This resulted in the inclusion of nine articles.

### 3.2. Study Characteristics 

Characteristics of the app intervention studies included in this review are presented in Table A1 in Appendix A. Five studies were conducted in North America [35,36,37,38,40], one study in Australia [34], one study in Europe [33], one study in Turkey [21] and another one in Spain [41]. Nine studies were randomized controlled trials (*n* = 9) with 2-group [21,33,34,35,36,37,40,41] or 3-group [38] study designs. 

Of the nine included studies (*n* = 1495 participants), 1188 participants (79.5%) were female. Seven studies were carried out in mixed gender populations [21,33,34,35,36,40,41]. Two studies were carried out among female participants only [37,38]. One study targeted pregnant women between 10 and 20 weeks of gestation [37].

Nine mobile health interventions were designed to increase overall daily PA among the inactive group. Six studies were physical activity interventions alone [21,33,34,36,38,40], and both PA and SB were targeted in two studies [35,37,41]. One study targeted PA, quality of life, self-efficacy, and exercise motivation for the inactive group [21].

Two studies involved interventions delivered solely via a mobile application (stand-alone intervention) [36,40] and seven studies [21,33,34,35,37,38,41] involved interventions that used both mobile apps and other intervention strategies (multi-component intervention), such as physical education sessions, counseling sessions, information pamphlet, motivational emails, online community and pedometer. 

The duration and intensity of the intervention in included studies varied. The intervention time ranged from 3 weeks [33] to 6 months [38]. The type of control groups also varied. One study used a wait-list control [34], two studies used a non-intervention control group [21,35] and three studies provided their control group with basic health information and instructions [33,36,41]. One study compared the intervention group providing the physical promotion app and diet app with the control group providing a diet app alone [40]. Two studies provided their control group with the accelerometer compared with accelerometer and app for the intervention group [37,38].

A variety of PA measurement tools were used. One study used more than one measurement tool [40]. Eight studies used objective measures including: accelerometer [35,38,40], pedometer [34], smart band [33,37,41] and smartphone [21,36]. Subjective questionnaires were used in three studies [21,34,37,41].

### 3.3. Risk of Bias 

**Figure 2:**
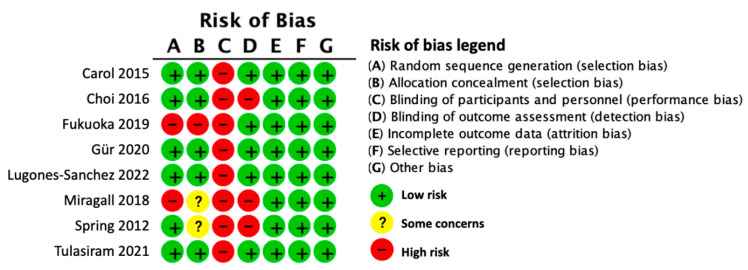
Risk of bias summary.

The assessment for each risk of bias item across all included studies is presented in Figure 2. All studies carried a high risk of in participants’ personal blinding owing to the nature of the interventions [21,33,34,35,36,37,38,40,41]. For the selection bias, two studies [33,38] lacked an explanation for how they generated the random sequences. Allocation concealment bias was noted in three studies [33,35,38]. Two of them did not mention the allocation process, which implies some concerns [33,35]. Three studies were considered to have a high risk of bias in blinding the outcome assessment [33,35,37].

### 3.4. Effect of Intervention

Nine studies [21,33,34,35,36,37,38,40,41] examined the effects of mHealth intervention on PA; One study was excluded because the required information was not available [40]. Data were pooled from eight interventions for meta-analysis. The result of the meta-analysis was statistically significant and favored mHealth interventions (MD = 8.72, 95% CI = 2.34 to 15.10). Four studies [33,34,35,41] reported the follow-up effects of mHealth intervention on physical activity; the meta-analysis result of the four pooled studies was statistically significant and favored mHealth interventions (MD = 14.54, 95% CI = −2.25 to 31.34).

**Figure 3:**
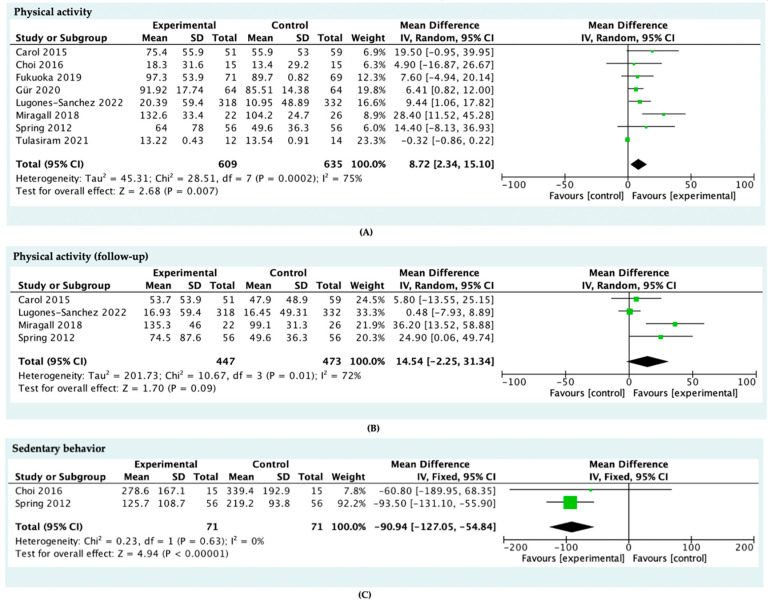
Meta-analysis of the effect of mobile health applications among the inactive population on (**A**) physical activity, (**B**) physical activity follow-up, and (**C**) sedentary behavior.

Two studies [35,37] examined the effects of mHealth intervention on SB; all studies were included in the meta-analysis (see Figure 3). Data were pooled from two interventions for meta-analysis. The result of the meta-analysis was statistically significant and favored mHealth interventions (MD = −90.94, 95% CI = −121.05 to −54.84).

### 3.5. Study Quality 

The quality assessment result of the included studies can be found in Table A2 in Appendix B. The quality of included studies ranged from low (*n* = 1) [33] to mid (*n* = 1) [35] and high (*n* = 7) [21,34,36,37,38,40,41]. Most included studies fulfill the CONSORT criteria to provide a strong scientific rationale and described their participant eligibility, statistical methods and interventions clearly. Few studies reported sample size calculations [21,36,38,40,41] (*n* = 5) and included blinding procedures in their study design (*n* = 2) [34,40].

## 4. Discussion

The systematic review and meta-analysis were conducted to quantify the reliable evidence about the impact of mHealth interventions on the PA promotion and behavior modification among inactive individuals. The reviewed studies delivered interventions by means of mobile applications. The duration of the intervention varied from 3 weeks to 6 months. Our results revealed that interventions using mHealth could strongly increase the PA level and reduce the SB among the inactive participants. 

In general, some studies [35,36,40,41] concluded that mHealth is effective in promoting exercise among inactive people, the results of the meta-analysis reported in this article also confirmed the observable utility of mHealth intervention. Furthermore, the reliability of our conclusions is enhanced by the results of our study quality assessment (Table A2 in Appendix B). As shown in Figure 3, the PA time of the intervention group increased on average by 8.72 min of PA per day. In other words, this meant an inactive individual could obtain an increase of 61.04 min on PA each week, which accounted for nearly 40 percent of the recommendation level [2]. Additionally, four studies reported long-term follow-up measures of PA [33,34,35,41]. The magnitude of the increase reached 14.54 min per day, which demonstrated the beneficial effect could be nicely sustained in the long run. This contradicted the result from a relevant study that argued people can hardly keep a modified healthy lifestyle for more than six months [42]. One possible explanation is that the use of multiple components in the reviewed studies led to better outcomes [43].

The increase in PA time appeared to be more prominent post-intervention according to this meta-analysis. It must be noted that a small proportion of studies in our paper reported follow-up measures for enhancing PA, highlighting the lack of evidence for a long-term increase in PA. Therefore, it is not possible to evaluate the long-term effectiveness of mHealth intervention. This lack of evidence in examining the effectiveness of long-term follow-up is seen in other reviews [26,44,45].

Given clinical recommendations suggest ongoing behavioral support is necessary for lifestyle changes to be sustained [6,7,8,9], continuous use of mHealth apps could make this feasible and cost-effective. In terms of the generalizability of these results, participants included in reported studies were male and female adults, with a BMI indicating over-weight or obesity, predominantly from the U.S. or other developed countries and occasionally with a diagnosed disease, such as diabetes. Results could therefore be generalized to clinical populations such as diabetes mellitus or osteoarthritis patients [46,47], and more research would be required in developing countries. In addition, the limited sample size could influence the reliability of our conclusion. Given the fact that prolonged behavior change could bring out health benefits [31] and improve other dimensions of physical fitness such as BMI and weight, the behavior change therefore requires a relatively long-term observation [43,48,49]. Hence, future research that studies long-period health behavior change is highlighted.

There is a study [12] suggesting that increased PA does not necessarily lead to improved SB when using traditional face-to-face intervention methods, while the results of the meta-analysis in this paper show synergistic benefits of mHealth for the increase in PA and the decrease in SB. It was shown that interventions targeting ease of SB present a mean reduction of 90.94 min in SB time. The result was inspiring since the evidence indicated that just 30 min of SB reallocated to light PA could deliver clinically considerable health outcomes [5]. Compared with another meta-analysis focusing on an ordinary group [31], the result of inactive people within this review showed an additional reduction of 45.94 min per day in SB time. We speculated that the daily sedentary feature of the inactive group led to this extra change in the SB ease effects. Thus, the inactive group deserves specific analysis when conducting studies at a large scale involving a huge number of ordinary people on physical activity and sedentary behavior in order to avoid its misleading impacts on the results. In addition, different inclusion criteria and the sample size could also be considered as one of the reasons. Based on the above discussions, we propose the following suggestions. First, future research could further evaluate the relationship between PA and SB, such as building up accurate quantitative models. Additionally, further exploration is required to gather data from long-term interventions on SB to assess the potentially retentive ability of mHealth in the reduction effect on the SB.

The present findings may present practical implications for mHealth intervention in the future. Long-term user engagement and solid theoretical foundation for mHealth intervention are required for achieving the improved PA. However, the effectiveness of different behavior change theories could not be examined in the present review since limited intervention theory information was given in the included studies. It is likely that intuitive app-use can have a better influence on enhancing the exercise level of inactive individuals. Together with the findings, we hereby propose recommendations for better practical effects of mHealth interventions. First, mHealth providers can develop more functions to improve the app adherence. For example, social participation can be included when designing an mHealth solution. Second, some behavior intervention techniques could be implemented to the mHealth solutions. Examples include “social support”, “hints”, and “goal setting”. In this study, there was a lack of clear and consistent reporting on what behavior change techniques were used in the intervention. The reporting of intervention content should be improved for assessing the effectiveness of the behavior intervention techniques.

## 5. Limitation 

This study exhibits some limitations and therefore calls for future studies. First, this systematic review was not registered on PROSPERO, which is considered a major limitation of our study. However, the search strategy, study selection, and quality assessment were carried out in accordance with established guidelines. Multiple researchers participated in this review to ensure the accuracy of the data and the credibility of the results. We also conducted a meta-analysis of all studies, along with a summary of the risk of bias for all studies. However, there is still a risk that bias could be diluted in the discussion and conclusions of this review. This risk can be reduced by assessing the quality of evidence for each outcome, for example, using the GRADE system. Second, the samples of this study were dominated by females. The gender differences cannot be ignored in the analysis since the characteristics of males and females vary, which could result in diverse behavior habit, schedule flexibility, and long-term mental status. Future research should control variables and make the male–female ratio more balanced to ensure the results reflect the impact of the intervention on general individuals. Plus, multi-group analysis for males and females is appreciated as well. Third, when appraising the same outcome categorized in our study, over one indicator was adopted. Thus, we faced the difficulty of selecting and making a consensus on the best-suited measurement. A further limitation is mainly relevant to the duration—most of the included studies lasted less than eight weeks. As a result, the long-term impact cannot be observed. Finally, only one study referred to the related theoretical frameworks, and thus, we might not identify its concrete role in the context of this study.

## 6. Conclusions

In conclusion, the interventions using mHealth could well accommodate the requirements of increasing PA and reducing SB among a specific population—inactive individuals. Future studies could follow our research approach to explore the long-term effect on mental health and the benefits of homogeneous methods on the pretext of mHealth in the light of the overall health of the inactive. In practice, our results could provide suggestions for the functions of mHealth to better improve physical activity and mitigate sedentary behavior.

## Figures and Tables

**Table 1:** PICO for research questions.

Concept	Description of Detail
Population	Inactive Population
Intervention	Mobile health intervention
Comparison	Control group without the mobile health intervention
Outcomes	Effects of mobile health intervention on physical activity and sedentary behavior

**Table 2:** Major search terms.

Concept	Description of Detail
Intervention	Application OR App OR Smartphone OR Smart Phone
Outcomes	Sedentary OR sedentary behavior OR sedentary behavior OR sitting OR screen time OR inactive OR inactivity

**Table 3:** List of search strings in different digital library.

Digital Library.	Query String	Scope	Limitation
Web of science	AB = (application OR app OR smartphone OR smartphone OR tablet) AND AB = (sedentary OR sedentary behavior OR sedentary behavior OR sitting OR screen time OR inactive OR inactivity)	Abstract	Limited to journals, English, 2006–2022
CINAHL	AB = (application OR app OR smartphone OR smartphone OR tablet) AND AB = (sedentary OR sedentary behavior OR sedentary behavior OR sitting OR screen time OR inactive OR inactivity)	Abstract	Limited to journals, English, 2006–2022
Scopus	(TITLE-ABS-KEY (application OR app OR smartphone OR smart AND phone OR tablet) AND TITLE-ABS-KEY (sedentary OR sedentary AND behavior OR sedentary AND behavior OR sitting OR screen AND time OR inactive OR inactivity)) AND PUBYEAR > 2006 AND (LIMIT-TO (PUBSTAGE, “final”)) AND (LIMIT-TO (DOCTYPE, “ar”) OR LIMIT-TO (DOCTYPE, “cp”)) AND (LIMIT-TO (LANGUAGE, “English”))	Title, Abstract and keywords	Limited to journals, English, publication stage: final, 2006–2022
PsychInfo	(application OR app OR smartphone OR smartphone OR tablet).tw. AND (sedentary OR sedentary behavior OR sedentary behavior OR sitting OR screen time OR inactive OR inactivity).tw.	Title and Abstract	Limited to journals, English, human, 2006–2022
PubMed	(application [Title/Abstract] OR app [Title/Abstract] OR smartphone [Title/Abstract] OR smartphone [Title/Abstract] OR tablet [Title/Abstract]) AND (sedentary [Title/Abstract] OR sedentary behavior [Title/Abstract] OR sedentary behavior [Title/Abstract] OR sitting [Title/Abstract] OR screen time [Title/Abstract] OR inactive [Title/Abstract] OR inactivity [Title/Abstract])	Title and Abstract	Limited to journals, English, human, 2006–2022

## Data Availability

All the data generated during this study is provided in the main manuscript.

## Appendix A

**Table A1:** Synthesis of main variables collected from RCTs (*n* = 9).

Study	Study Design	Duration	Participant Characteristic	Setting	Intervention	Dependent Variables	Outcome Measure	Comparison Group
[36]	RCT	Intervention exposure: 4 weeks	N = 26 Age 18~25 years Male (7) and Female (19) USA	Home	The SmPh app allows step tacking	App impact on cardiorespiratory fitness of college-going adults	Outcome: (i) Aerobic capacity; (ii) Ventilatory equivalent of carbondioxide (iii) Anaerobic threshold (iv) Treadmill distance and time; (v)Heart rate	Traditional walking prescription
[40]	RCT	Intervention exposure: 8 weeks	N = 95 Age >45 years Male (26) and Female (69) USA	Home	Three mobile apps shape the user towards more physical activity and fewer daily sedentary time	App impact on daily activity and sedentary time	Outcome: (i) Physical activity (i.e., accelerometer-derived moderate-to-vigorous physical activity) and (ii) Sedentary behavior (i.e., accelerometer-derived sedentary time, EMA-derived sitting time)	A diet-tracking control app
[35]	2-group RCT	Intervention exposure: 3 week, 20 week follow-up	N = 204 Age 21~60 years Male (48) and Female (156) USA	Home	(i) Behavior treatment; (ii) Handheld tool to record and self-regulate their targeted behaviors	The effect of Remote coaching supported by mobile tech- nology and financial incentives to improve diet and activity.	Outcome: (i) Fat and fruit/vegetable consumption; (ii) the saturated fat goal: the Harris–Benedict equation; (iii) Minutes of physical and sedentary activity	No control group
[33]	2-group RCT	Intervention exposure: 3-week intervention and a 3 months follow-up	N = 76 Age 18~40 years Female (65), Male (11) UK	Community, workplace home	(i) Fitbit One to measure steps and provides motivational messages; (ii) Internet-based motivational intervention	The effect of an Internet-based motivational intervention supported by pedometers on physical activity	Outcome: (i) Physical activity; (ii) Stages of Change Questionnaire for exercise; (iii) Decision Balance Questionnaire for exercise; (iv) Self-Efficacy Questionnaire; (v) Processes of Change Questionnaire	Without Fitbit one and internet-based motivational intervention
[34]	2-group RCT	Intervention exposure: 50-day, 20 week follow-up	N = 76 Age 18~65 years Female (82), Male (26) Australia	Home	(i) Active Team app to encourage friendly rivalry within friendship group; (ii)A pedometer to measure steps	The effect of an online social networking physical activity intervention with pedometers delivered via Facebook app	Outcome: Physical activities (Active Australia Survey, Assessment of Quality of Life-6D (AQoL-6D) scale, 36-item Short Form Health Survey)	Teams allocated to the control condition were placed on a waiting list to receive access to the intervention (app and pedometer)
[37]	2-group RCT	Intervention exposure:12 weeks	N = 30 Age 30~36 years pregnant women between 10 and 20 weeks of gestation USA	Home	(i) Initial Brief In-Person Session; (ii) Mobile phone app plus Fitbit	The effect of mobile health intervention in promoting physical activity in Pregnant women	Outcome: Physical activities (The Stanford Brief Physical Activity Survey) Other measures: (i) The Self-Efficacy for Physical Activity; (ii) survey, quiz, scale and checklist	Fitbit Ultra only (accelerometer)
[38]	3-group RCT	Intervention exposure:3 months	N = 210 Age 40~60 years Female (210) USA	Home	Use the app and accelerometer for 9 months	The effects of APP on levels of physical activity	Outcome: Physical activity Other measures: (i) Survey, quiz, scale	Control group: use accelerometer for 9 months
[21]	2-group RCT	Intervention exposure: 8 weeks	N = 128 Age 19–26 years Female (112), Male (16) Turkey	Home	(i) ERVE smartphone app; (ii) An educational video each week under the exercise education component of the ap; (iii) Researchers sent a message once a week to increase motivation.	The effects of a smartphone app on physical activity, quality of life, self-efficacy, and exercise motivation for inactive people	Primary outcome: self-efficacy, health-related quality of life, and motivational orientation for exercise Secondary outcome: BMI and levels of physical activity	No intervention
[41]	2-group RCT	Intervention exposure: 3 months	N = 650 Age 20–65 years Female (445) Male (205) Spain	Primary care center	(i) 5 min of lifestyle counseling before randomization; (ii) low-intensity intervention consisting of a smartphone with the EVIDENT 3 app and a smart band for 3 months;	The effects of a smartphone app combined with a smart band on weight loss, physical activity, and caloric intake in a population with overweight and obesity	(i) Body weight; (ii) Physical activity: International Physical Activity Questionnaire–Short Form; (iii) Caloric intake (kcal/day) and dietary habits: semiquantitative Food Frequency Questionnaire.	A brief counseling only

## Appendix B

**Table A2:** Synthesis of quality assessment of included RCTs (*n* = 9).

	[36]	[40]	[35]	[33]	[34]	[37]	[38]	[21]	[41]
Title and abstract									
(a) identification as randomized trial in title; (b) structured summary	1	1	1	0.5	1	1	1	1	1
Introduction									
(a) scientific background/rationale; (b) specific objectives/hypotheses	1	1	1	1	1	1	1	1	1
Methods									
Trial design: (a) description of trial design; (b) changes in methods after trial commencement	0.5	0.5	0.5	0.5	0.5	0.5	0.5	0.5	0.5
Participants									
(a) eligibility criteria; (b) settings and locations of data collection	1	1	1	1	1	1	1	1	1
Interventions									
Descriptions of sufficient details to allow replication	1	1	1	1	1	1	1	1	1
Outcomes									
(a) pre-specified primary and secondary outcomes; (b) changes to outcomes after trial commencement	0.5	0.5	0.5	0.5	0.5	0.5	0.5	0.5	0.5
Sample size									
(a) how sample size was determined; (b) if applicable, interim analysis/stopping guidelines	0.5	0.5	0	0	0	0	0.5	0.5	0.5
Randomization—sequence generation									
(a) method used; (b) type of randomization including any type of restriction	0.5	1	1	0	0.5	0.5	0	0.5	0.5
Allocation concealment mechanism									
Implementation of random allocation sequence, including concealment	1	1	1	0	1	1	0	1	1
Implementation									
Who generated random allocation sequence, who enrolled participants, who assigned participants	0	1	0	0	0.5	1	0	1	1
Blinding									
(a) if done, who was blinded and how; (b) if relevant, similarity of interventions	0	0.5	0	0	0.5	NA	NA	NA	NA
Statistical methods									
Statistical methods used (a) for primary outcomes; (b) additional analyses	1	1	1	1	1	1	1	1	1
Results									
*Participant flow* (a) number of participants randomized, receiving treatment, and analyzed; (b) losses and exclusions, with reasons	1	1	1	0.5	1	1	1	1	1
Recruitment									
(a) dates of recruitment and follow-up; (b) why the trial ended	0.5, NA	0.5, NA	0.5, NA	0.5,NA	0.5,NA	0.5,NA	0.5,NA	0.5,NA	0.5,NA
Baseline data									
A table with baseline demographic and clinical characteristics for each group	1	1	1	0	1	1	1	1	1
Numbers analyzed									
For each group, number of participants included in each analyses	1	1	1	1	1	1	1	1	1
Outcomes and Estimation									
(a) results for each group, and the estimated effect size and its precision; (b) absolute and relative effect sizes for binary outcomes	0.5, NA	0.5, NA	0, NA	0, NA	0.5, NA	0.5, NA	0.5, NA	0.5, NA	0.5, NA
Ancillary analyses									
Results of any other analyses performed, distinguishing pre-specified from exploratory	1	1	0	0	1	0	0	0	0
Harms									
Harms or unintended effects in each group	0	1	0	0	0	1	1	0	0
Discussion									
Limitations Trial limitations/bias/multiplicity of analyses	1	1	1	1	1	1	1	1	1
Generalisability									
Generalisability (external validity/applicability) of findings	0	1	1	1	0	0	0	0	0
Interpretation									
Consistent with results and balanced	1	1	1	1	1	1	1	1	1
Other information									
RegistrationRegistration number and name of registry	1	1	0	0	1	0	1	0	1
Protocol									
Where full trial protocol can be accessed	0	1	0	0	1	1	1	0	1
Funding									
Sources of funding/ role of funders	0	1	1	1	1	0	1	1	1
Study quality score attainable	24	24	24	24	24	23	23	23	23
Study quality score	16	21.5	15.5	11.5	19	17	17.5	16	18
Study quality percentage	66.7	87.8	63.2	46.9	77.6	70.8	76.1	69.6	78.2
Study quality rating	High	high	mid	low	high	High	High	High	High
